# Effects of cytarabine on activation of human T cells – cytarabine has concentration-dependent effects that are modulated both by valproic acid and all-trans retinoic acid

**DOI:** 10.1186/s40360-015-0012-2

**Published:** 2015-05-02

**Authors:** Elisabeth Ersvaer, Annette K Brenner, Kristin Vetås, Håkon Reikvam, Øystein Bruserud

**Affiliations:** Institute of Clinical Science, University of Bergen, Bergen, Norway; Institute of Biomedical Laboratory Sciences, Bergen University College, Nygårdsgaten 112, P.O. Box 7030, N-5020 Bergen, Norway; Department of Medicine, Haukeland University Hospital, Bergen, Norway

**Keywords:** T cells, Cytarabine, All-trans retinoic acid, Valproic acid, Acute myeloid leukemia

## Abstract

**Background:**

Cytarabine is used in the treatment of acute myeloid leukemia (AML). Low-dose cytarabine can be combined with valproic acid and all-trans retinoic acid (ATRA) as AML-stabilizing treatment. We have investigated the possible risk of immunotoxicity by this combination. We examined the effects of cytarabine combined with valproic acid and ATRA on *in vitro* activated human T cells, and we tested cytarabine at concentrations reached during *in vivo* treatment with high doses, conventional doses and low doses.

**Methods:**

T cells derived from blood donors were activated *in vitro* in cell culture medium alone or supplemented with ATRA (1 μM), valproic acid (500 or 1000 μM) or cytarabine (0.01-44 μM). Cell characteristics were assessed by flow cytometry. Supernatants were analyzed for cytokines by ELISA or Luminex. Effects on primary human AML cell viability and proliferation of low-dose cytarabine (0.01-0.5 μM) were also assessed. Statistical tests include ANOVA and Cluster analyses.

**Results:**

Only cytarabine 44 μM had both antiproliferative and proapoptotic effects. Additionally, this concentration increased the CD4:CD8 T cell ratio, prolonged the expression of the CD69 activation marker, inhibited CD95L and heat shock protein (HSP) 90 release, and decreased the release of several cytokines. In contrast, the lowest concentrations (0.35 and 0.01 μM) did not have or showed minor antiproliferative or cytotoxic effects, did not alter activation marker expression (CD38, CD69) or the release of CD95L and HSP90, but inhibited the release of certain T cell cytokines. Even when these lower cytarabine concentrations were combined with ATRA and/or valproic acid there was still no or minor effects on T cell viability. However, these combinations had strong antiproliferative effects, the expression of both CD38 and CD69 was altered and there was a stronger inhibition of the release of FasL, HSP90 as well as several cytokines. Cytarabine (0.01-0.05 μM) showed a dose-dependent antiproliferative effect on AML cells, and in contrast to the T cells this effect reached statistical significance even at 0.01 μM.

**Conclusions:**

Even low levels of cytarabine, and especially when combined with ATRA and valproic acid, can decrease T cell viability, alter activation-induced membrane-molecule expression and decrease the cytokine release.

**Electronic supplementary material:**

The online version of this article (doi:10.1186/s40360-015-0012-2) contains supplementary material, which is available to authorized users.

## Background

Intensive anticancer therapy causes an acute and severe panleukopenia, including T lymphopenia, that may last for 2-4 weeks [1], and after hematopoietic reconstitution these patients usually develop a CD4^+^ T cell defect that may persist for several months especially in adults [2]. This persisting T cell defect has been described both after conventional chemotherapy as well as after autologous and allogeneic stem cell transplantation. All these three therapeutic strategies are used in the treatment of acute myeloid leukemia (AML), and early lymphocyte recovery after such intensive treatment then predicts superior relapse-free survival. This observation suggests that early immunological events [3-6] and possibly also cancer- or AML-related inflammation [7] are important for AML cell survival and proliferation after chemotherapy.

Even though AML is an aggressive malignancy and intensive chemotherapy eventually in combination with stem cell transplantation is the most effective antileukemic therapy [8], many elderly or unfit patients cannot receive this treatment due to an unacceptable risk of severe toxicity and early treatment-related mortality. These patients will either receive supportive care alone or in combination with low-toxicity AML-stabilizing chemotherapy [8]. One such low-toxicity AML stabilizing chemotherapy is single-drug, low-dose subcutaneous cytarabine injections usually administered as daily treatment for 10 days with 4-6 weeks intervals; this treatment can eventually be combined with oral all-trans retinoic acid (ATRA) and valproic acid, i.e. a histone deacetylase inhibitor [9-14]. Previous *in vivo* clinical studies have shown that the triple combination of low-dose cytarabine, ATRA and valproic acid has immunomodulatory effects through a normalization of the increased pre-therapy levels of circulating Treg cells, whereas the levels of Th17 cells are not affected by the treatment [15]. However, very little is known both about the acute and long-term effects of such treatment on the T cell system and whether T cell toxicity affects its antileukemic efficiency. In the present study we therefore investigated the *in vitro* effects of various cytarabine concentrations, valproic acid and ATRA on activated T cells.

## Methods

### Cell donors and preparation of peripheral blood mononuclear cells

The studies were approved by the local Ethics Committee (Regional Ethics Committee III, University of Bergen, Bergen, Norway) and buffy coats were derived from healthy blood donors after informed consent. Peripheral blood mononuclear cells (PBMC) were isolated by density gradient separation (Ficoll-Hypaque; NyCoMed, Oslo, Norway; specific density 1.077) from buffy coats from seven healthy blood donors (median age 29 years; 3 male and 4 female). Viability, proliferation and cytokine release was examined for all individuals, CD4:CD8 ratio and expression of activation markers were investigated only for 3 randomly selected individuals.

### Drugs

Cytarabine (Cytosine β-D-arabinofuranoside; Sigma-Aldrich, USA) was dissolved in ddH_2_O to obtain a concentration of 400 μM before aliquoted, ATRA (Sigma-Aldrich; Oslo, Norway) was dissolved in 96% ethanol to 1 mM and valproic acid (Desitin Arzneimittel GmbH, Hamburg, Germany) was diluted in saline to 60 mM. All drugs were stored at -80°C. Drugs were thawed on the same day they were used in experiments and based on previous studies of *in vivo* levels the drugs were tested at the following concentrations that are relevant to low-toxicity AML treatment: valproic acid 1000 μM and 500 μM [16], cytarabine 0.35 μM and 0.01 μM [17-19], and ATRA 1 μM [20-22]. Cytarabine was also tested at 44 μM and 1 μM corresponding to high-dose therapy [23,24]. The relevance of these 4 cytarabine concentrations with regard to the levels reached *in vivo* is discussed in detail below in the Discussion section.

### Cell culture

PBMC were suspended in pre-warmed X-Vivo 10® medium (BioWhittaker, Cambridge, MA, USA) with 10% FBS (Lonza Braine, Belgium) and cultured in 24-well culture plates at a final concentration of 0.5 × 10^6^ cells/mL (viability and proliferation analyses) or 1 × 10^6^ cells/mL (analysis of activation markers). T lymphocytes were activated by 0.6 μg/mL of mouse anti-human CD3 (Pelicluster, Amsterdam, The Netherlands) and 0.4 μg/mL of mouse anti-human CD28 (Pelicluster). Drugs were prepared from frozen stock solutions the same day as the experiments. Cultures were incubated at 37°C in a humidified atmosphere of 5% CO_2_ before cells/supernatants were harvested.

### Flow cytometric analysis of viability, proliferation and membrane molecule expression

Flow cytometry was performed by FACS Canto II. For each sample at least 20 000 CD5^+^ lymphocytes were counted. All results were analyzed by FlowJo software (Tree Star, Inc., OR, USA).

#### Proliferation and viability assay

PBMC dissolved in PBS were stained strictly according to the manufacturer’s instructions in the CellTrace Violet Cell Proliferation Kit (Invitrogen); thereafter cells were washed and cultures prepared as described above. The cells were harvested after 4 days and washed in ice-cold PBS before being resuspended in Annexin V Bindings buffer (BD Biosciences, Trondheim, Norway) and stained for 15 minutes with LIVE/DEAD Far Red Fixable Dead Cell Stain (Invitrogen, Oregon, USA). Annexin V conjugated with Alexa488 (Invitrogen, Oregon, USA) and anti-human CD5 conjugated with PE-CY7 (clone L17F12; BD) was added, cells were further incubated for 15 minutes and thereafter washed in ice-cold 1% BSA/PBS before four-color flow cytometric analysis.

#### Analysis of CD4/CD8 ratio and activation marker expression

Cells were harvested after 20, 44 and 68 hours of culture, thereafter washed in ice-cold 1% BSA/PBS followed by 10 minutes of incubation in 200 μg/ml of Fc-receptor blocking agent (Octagam, Octapharma Ltd, Coventry, UK) before the following anti-human antibodies were added; FITC-conjugated anti-CD5 (L17F12; BD Biosciences), V500-conjugated anti-CD8 (RPA-T8; BD Biosciences), PerCPCy5.5-conjugated anti-CD4 (RPA-T4; BD Pharmingen), PE-conjugated anti-CD25 (M-A251; BD Biosciences), PE-CY7-conjugated anti-CD69 and APC-conjugated anti-CD38. Cells were incubated with antibodies on ice for 20 minutes, washed once in 1% BSA/PBS and finally analyzed by flow cytometry.

### Analysis of soluble mediator concentrations

#### Luminex analyses

Culture supernatants were harvested after 4 days and stored at -80°C until analyzed. Cytokine levels were determined by Human Cytokine Panel A Fluorokine® Multianalyte Profiling (MAP) Kit (LUH000; R&D Systems, Abingdon, UK). All analyses were performed strictly according to the manufacturer’s instructions. Standard curves were constructed by using the mean of duplicate determinations, and differences between duplicates were generally <10% of the mean. The minimal detectable levels were IFNγ 1.27 pg/mL, TNF-α 1.5 pg/mL, G-CSF 1.48 pg/mL, GM-CSF 1.98 pg/mL, VEGF 1.84 pg/mL, bFGF 4.91 pg/mL, IL1RA 10.91 pg/mL, IL1α 0.36 pg/mL, IL1β 0.57 pg/mL, IL2 2.23 pg/mL, IL4 4.46 pg/mL, IL5 0.71 pg/mL, IL6 1.11 pg/mL, IL8 1.97 pg/mL (CXCL8), IL10 0.30 pg/mL, IL17 1.1 pg/mL, CCL2 (MCP-1) 0.47 pg/mL, CCL3 (MIP-1α) 1.45 pg/mL, CCL4 (MIP-1β) 0.74 pg/mL, CCL5 (RANTES) 1.91 pg/mL, CXCL5 (ENA-78) 4.14 pg/mL.

#### Enzyme-linked immuno-sorbent analyses (ELISA)

Supernatants were harvested and stored as described above. Mediator levels were determined by HSP90α human EIA kit (ADI-EKS-895; Enzo Life Sciences, Exeter, UK), HSP70 high sensitivity ELISA kit (ADI-EKS-715; Enzo Life Science) TRAIL/TNFSF10 immunoassay (Quantikine, R&D Systems), human CD95/Fas Ligand/TNFSF6 immunoassay (Quantikine, R&D Systems). All analyses were performed strictly according to the manufacturer’s instructions. Standard curves were constructed based on the mean of duplicate determinations, and differences between duplicates were generally <10% of the mean. The minimal detectable levels were HSP90 0.05 ng/mL, HSP70 0.09 ng/mL, FasL 2.26 pg/mL, TRAIL 2.86 pg/mL.

### AML patient samples and analyses

The effect of low-doses cytarabine were also tested on primary human AML cells from 48 consecutive patients (median age 67 years; range 24-83 years; 23 female and 25 male). Our strategy for recruitment of consecutive patients has been described in detail previously [25]. All patients were tested at the time of diagnosis before they eventually received antileukemic treatment. The AML cells were isolated from peripheral blood using density gradient separation (Lymphoprep; Axis Shield, Oslo, Norway) and contained at least 90% blasts. For cell viability measurement, AML cells (2 × 10^5^ cells/well) were incubated in flat-bottomed microtiter plates (200 μl/well; Costar 3596 culture plates; Costar, Cambridge, MA, USA) in Stem Span SFEM™ medium (Stem Cell Technologies; Vancouver, BC, Canada) at 37°C in a humidified 5% CO_2_ incubator for 40 hours before the percentage of viable cells was determined by flow cytometry after staining with propidium iodide (PI) and Annexin V. Furthermore, for the proliferation assay 5 × 10^4^ AML cells/well were cultured in flat-bottomed microtiter plates (200 μl/well) in Stem Span for six days prior to addition of 37 kBq of ^3^H-thymidine (Perkin Elmer) to each well and incubated for 18 hours before cells were harvested and radioactive activity determined. The medium used in the proliferation assay was supplemented with the growth factors (20 ng/ml) G-CSF, SCF and Flt3l.

### Statistical analyses

Statistical comparisons were made by using GraphPad PRISM (version 5.0, GraphPad Software, Inc., USA) with repeated measures ANOVA (within-subjects ANOVA or ANOVA for correlated samples) with Dunnett’s Multiple Comparison Test (post-test). Differences were regarded as significant when p <0.05. Cluster analyses were performed by the use of J-Express 2011 analysis suite (MolMine AS, Bergen, Norway). The AML cell data were analysed with the IBM Statistical Package for the Social Sciences (SPSS) version 21 using pair sampled t-test and the Wilcoxon signed rank test to compare drug-containing and drugfree cultures.

## Results

### ATRA and low doses of valproic acid do not affect viability and proliferative capacity of activated normal T cells, whereas cytarabine has a dose-dependent antiproliferative and proapoptotic effect

**Figure 1:**
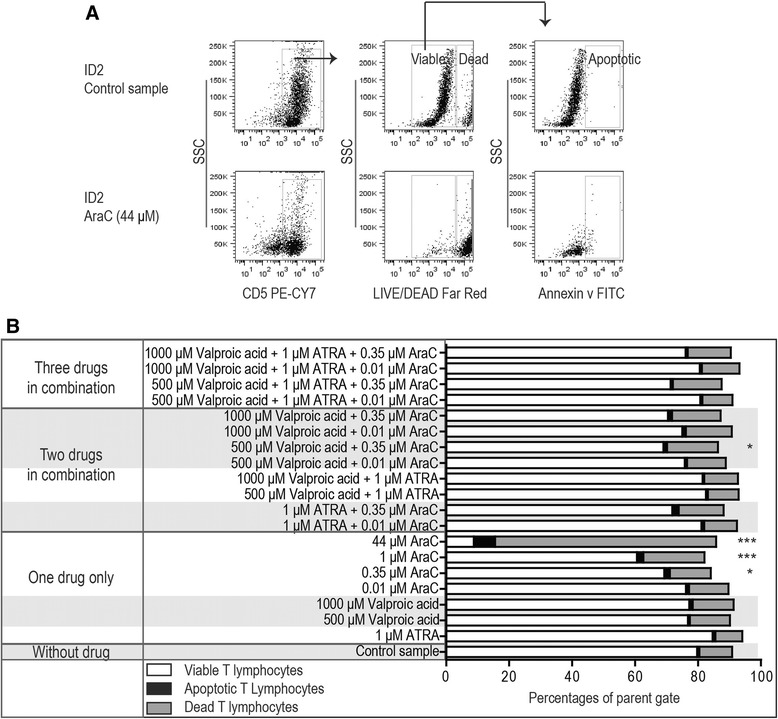
Viability of activated T lymphocytes after exposure to cytarabine, ATRA and valproic acid alone or in combinations. PBMCs derived from healthy blood donors (n = 7) were cultured *in vitro* with T cell activating anti-CD3 and anti-CD28, and the viability was analyzed by flow cytometry after 4 days of culture in medium alone or with the indicated drugs. **(A)** The gating strategy to analyze the viability of CD5^+^ T lymphocytes is shown for a control sample and the corresponding sample of cells exposed to cytarabine 44 μM. T cells were defined as (i) viable when negative staining with the LIVE/DEAD Far Red Fixable Dead Cell Stain, (ii) apoptotic when being LIVE/DEAD Far Red Fixable Dead Cell Stain negative and Annexin V positive, and (iii) dead when being LIVE/DEAD Far Red Fixable Dead Cell Stain positive. **(B)** The overall results are summarized as stacked bar graphs for the control samples and samples exposed to the indicated drugs or drug combinations. The results are presented as the mean percentages for viable, dead and apoptotic CD5^+^ T cells. A repeated measure ANOVA with Dunnett’s Multiple Comparison Test was used to determine statistically significant differences (*p <0.05; **p <0.01; ***p <0.001).

**Figure 2:**
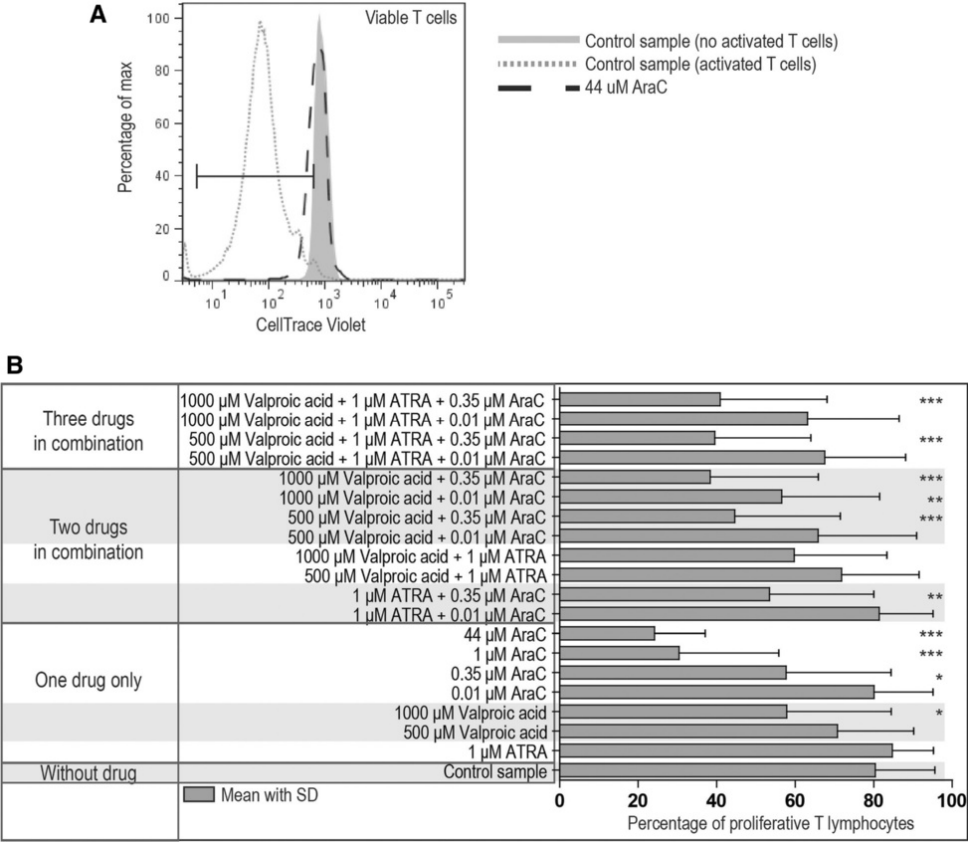
Proliferation of activated T lymphocytes after exposure to cytarabine, ATRA and valproic acid alone or in combinations. PBMCs derived from healthy blood donors (n = 7) were stained with the cell proliferation dye CellTrace™ Violet and subsequently activated by *in vitro* culture in the presence of anti-CD3 and anti-CD28. Flow cytometric analysis of proliferation was done after 4 days of culture in medium without drugs (control) or in the presence of drugs/drug combinations. **(A)** The gating strategy to measure the proliferative response of CD5^+^ T lymphocytes is shown for three representative samples (two control samples –unstimulated and stimulated– and one sample with cytarabine 44 μM). Cultures without anti-CD3 and anti-CD28 and thereby no proliferating cells were used as the negative gating control. **(B)** The overall results are presented as bar graphs for the control samples and samples exposed to the indicated single drugs or drug combinations. Results are presented as the mean percentages (with SD) of proliferative T cells. A repeated measure ANOVA with Dunnett’s Multiple Comparison Test was used to determine statistically significant differences (*p <0.05; **p <0.01; ***p <0.001).

PBMC derived from healthy blood donors (n = 7) were activated *in vitro* with anti-CD3 plus anti-CD28 during 4 days of culture in medium alone or medium supplemented with ATRA 1 μM, valproic acid 500 and 1000 μM or cytarabine 0.01-44 μM. The viability (Figure 1; Annexin-PI assay) and proliferation (Figure 2; the CellTrace Violet Cell proliferation assay) of CD5^+^ T cells were then analyzed by flow cytometry. ATRA and valproic acid (500 μM) did not cause any statistically significant alteration of T cell viability or proliferation. A small, but statistically significant, decrease in proliferation was detected after exposure to valproic acid (1000 μM). In contrast, cytarabine caused a dose-dependent reduction both in viability and proliferation. An increased fraction of apoptotic cells was then detected together with the decreased viability in the cytarabine-containing cultures; an observation suggesting that the decreased viability is caused by drug-induced apoptosis.

### Valproic acid increases the antiproliferative effect of cytarabine on normal human T cells

As described above, normal PBMC were activated in the presence of various drug combinations (Figures 1 and 2). Proliferation and viability was not altered for cultures containing various combinations of ATRA and valproic acid, and ATRA alone did not alter the antiproliferative or proapoptotic effects of cytarabine.

Valproic acid 500 and 1000 μM was also combined with cytarabine 0.01 and 0.35 μM; these cytarabine concentrations correspond to the *in vivo* levels reached during low-dose cytarabine treatment when the drug can be combined with ATRA and valproic acid [15,26,27]. Cytarabine 0.01 μM alone did not have any antiproliferative effect but a statistically significant antiproliferative effect was observed when cytarabine at this concentration was combined with valproic acid 1000 μM (Figure 2). Similarly, when cytarabine at a higher concentration of 0.35 μM was present together with valproic acid an increased antiproliferative effect was observed with valproic acid at both 1000 μM and 500 μM. Finally, dual or triple drug combinations did not have significant effects on T cell viability, the only exception being cytarabine 0.35 μM in combination with valproic acid 500 μM (Figure 1).

### The CD4:CD8 ratio is altered only by high-dose cytarabine but not by ATRA, valproic acid or low-dose cytarabine

**Figure 3:**
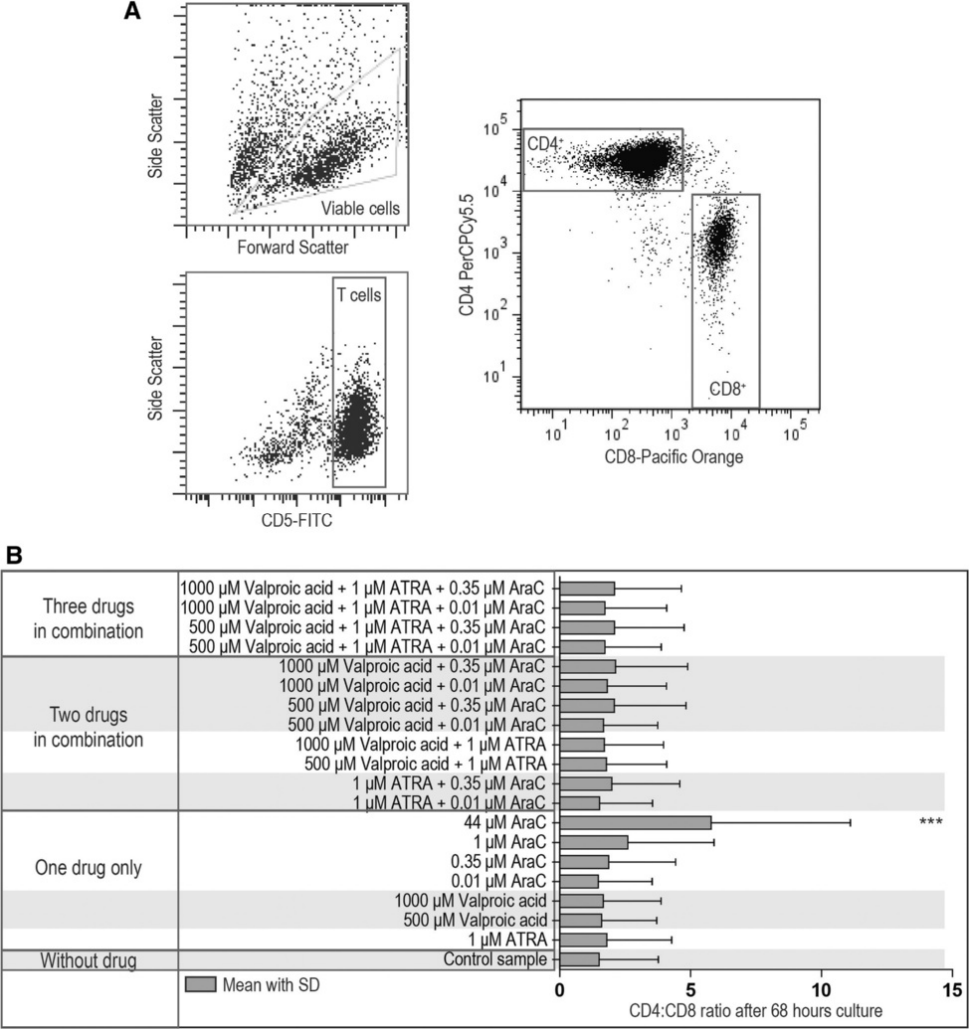
The CD4:CD8 ratio of activated T lymphocytes after exposure to cytarabine, ATRA and valproic acid alone or in combination. PBMCs derived from three healthy blood donors were activated by anti-CD3 plus anti-CD28 during 3 days of *in vitro* culture before flow cytometric analysis of the CD4:CD8 ratio. Cultures were prepared without drugs, with single drugs or with drug combinations. **(A)** The figure shows the gating strategy for estimation of CD4^+^CD5^+^ and CD8^+^CD5^+^ T cells lymphocytes in a representative experiment. **(B)** The overall results are presented as bar graph (mean ratio with SD) for the drugfree control cultures and cultures prepared with the indicated single drugs or drug combinations. A repeated measure ANOVA with Dunnett’s Multiple Comparison Test was used to determine statistically significant differences (*p <0.05; **p <0.01; ***p <0.001).

Normal PBMCs derived from 3 healthy individuals were activated as described above in the presence of various drug combinations. Only cytarabine (0.35, 1.0 and 44 μM) caused a dose-dependent reduction both in viability and proliferation. Proliferation, but not viability, was altered for cultures containing several of the dual or triple drug combinations. However, the CD4:CD8 ratio was significantly increased only by the highest cytarabine concentration of 44 μM whereas the ratio was not altered when the cells were cultured with single drugs at the other concentrations or any dual or triple drug combination (Figure 3).

### ATRA and valproic acid alters the expression of the CD69 and CD38 activation markers by CD4^+^ and CD8^+^ normal T cells whereas cytarabine has only minor effects

In our experimental model CD69 showed an expected high early expression after 20 hours of culture with anti-CD3 plus anti-CD28 both for CD4^+^ and CD8^+^ T cells, and thereafter it decreased gradually when tested after 44 and 68 hours. In contrast, CD38 showed a gradual increase for both T cell subsets during the same period after activation (3 healthy individuals tested).

**Figure 4:**
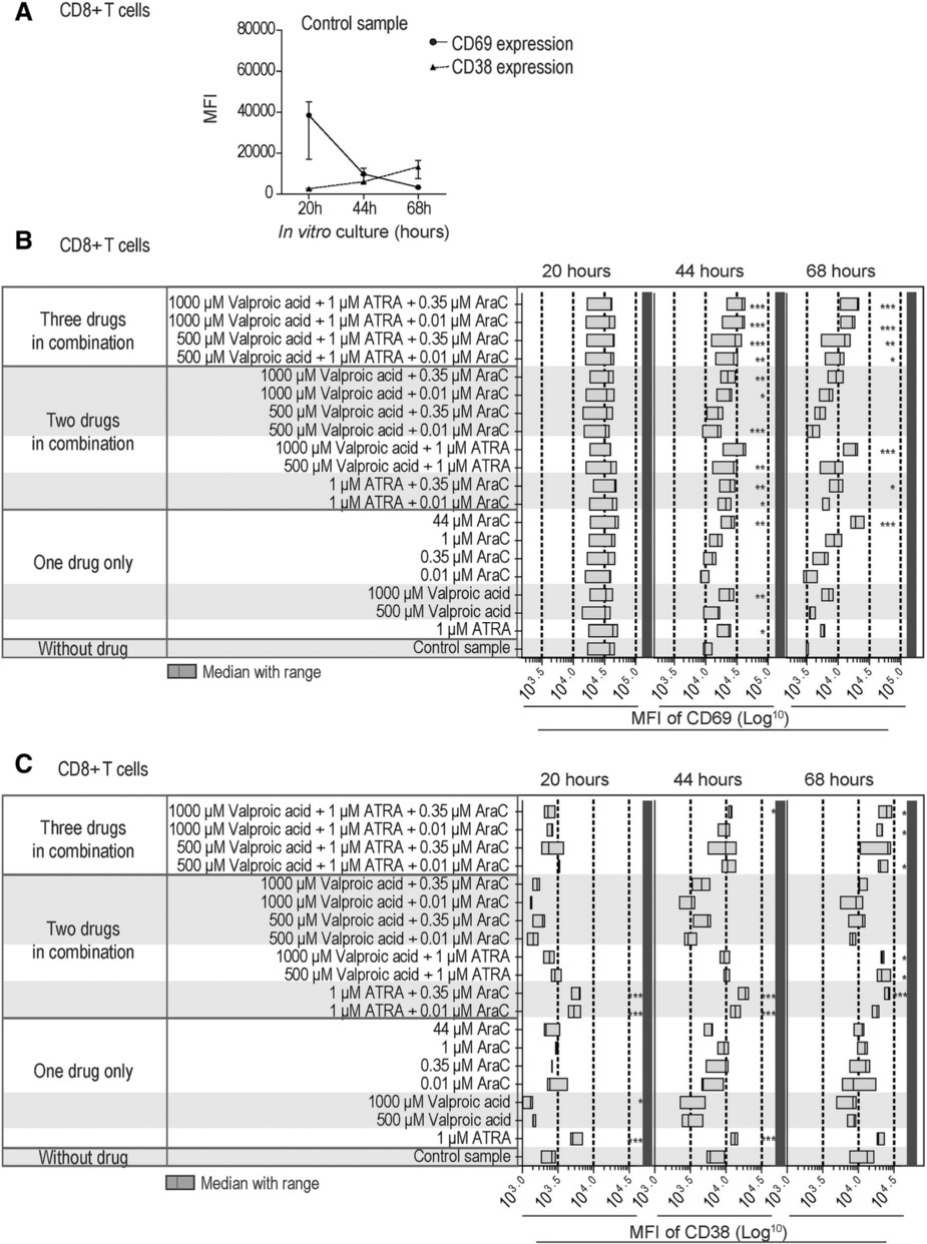
Expression of the CD69 and CD38 activation markers by anti-CD3 plus anti-CD28 activated CD8^+^ T lymphocytes – effects of cytarabine, ATRA and valproic acid tested alone or in combination. PBMCs derived from three healthy donors were activated during *in vitro* culture with anti-CD3 and anti-CD28, and flow-cytometric analysis of surface CD69 and CD38 expression was performed after 20, 44, and 68 hours of culture. **(A)** The figure shows the mean fluorescence intensity (MFI) of surface CD69 and CD38 expression by CD8^+^CD5^+^ T lymphocytes in drugfree control cultures after 20, 44, and 68 hours (median and range, three experiments). **(B)** The overall results for CD69 expression are presented as bar graph (median MFI and range) for CD8^+^CD5^+^ T lymphocytes cultured *in vitro* for 20, 44, and 68 hours in the presence of the indicated single drugs or drug combinations. **(C)** The overall results for CD38 expression are presented as bar graph (median MFI and range) for CD8^+^CD5^+^ T lymphocytes cultured *in vitro* for 20, 44, and 68 hours in the presence of the indicated single drugs or drug combinations. Repeated measures ANOVA with Dunnett’s Multiple Comparison Test was for the statistical analyses (*p <0.05; **p <0.01; ***p <0.001).

**Figure 5:**
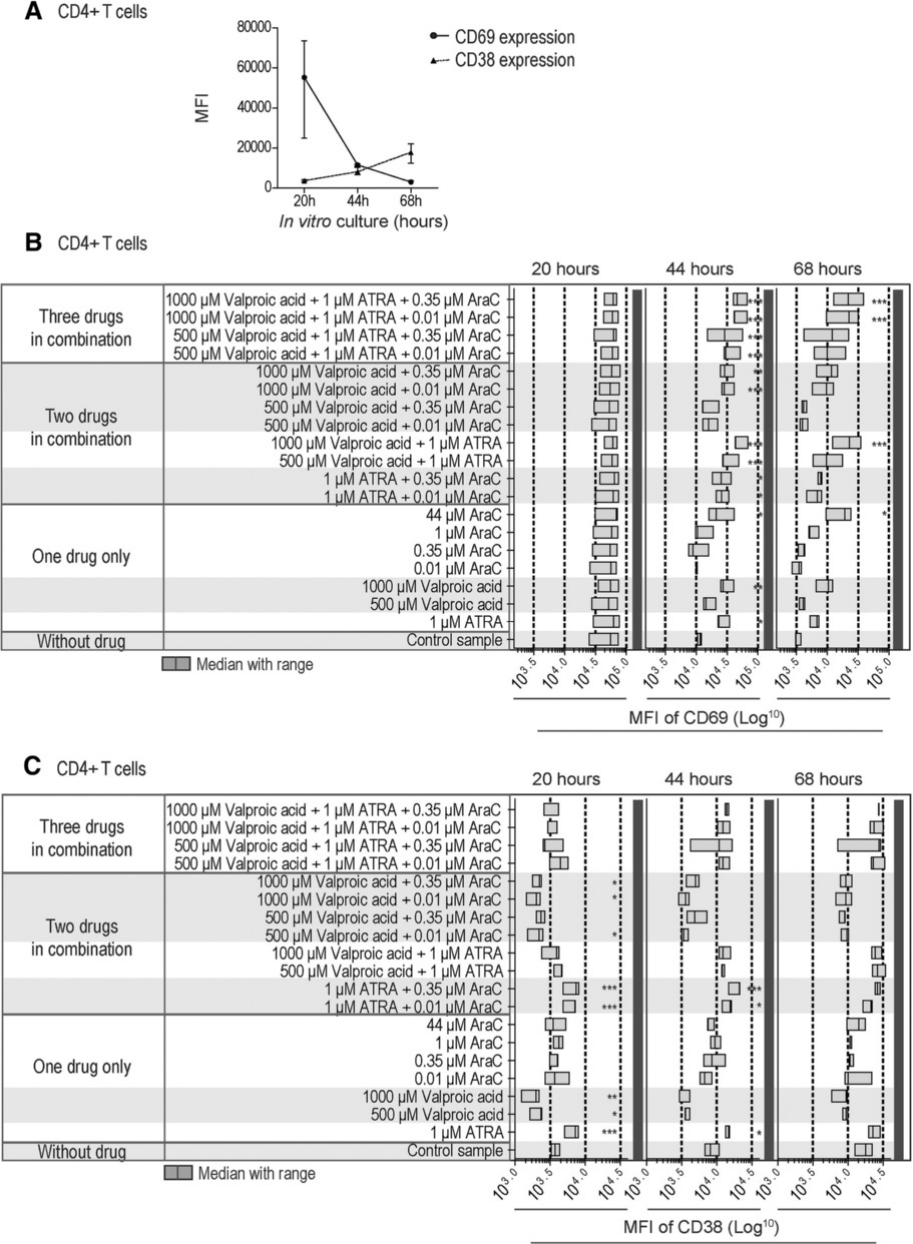
Expression of the CD69 and CD38 activation markers by anti-CD3 plus anti-CD28 activated CD4^+^ T lymphocytes – effects of cytarabine, ATRA and valproic acid tested alone or in combination. PBMCs derived from three healthy donors were activated during *in vitro* culture with anti-CD3 and anti-CD28, and flow-cytometric analysis of surface CD69 and CD38 expression was performed after 20, 44, and 68 hours of culture. **(A)** The figure shows the mean fluorescence intensity (MFI) of surface CD69 and CD38 expression by CD4^+^CD5^+^ T lymphocytes in drug-free control cultures after 20, 44, and 68 hours (median and range, three experiments). **(B)** The overall results for CD69 expression are presented as bar graph (median MFI and range) for CD4^+^CD5^+^ T lymphocytes cultured *in vitro* for 20, 44, and 68 hours in the presence of the indicated single drugs or drug combinations. **(C)** The overall results for CD38 expression are presented as bar graph (median MFI and range) for CD4^+^CD5^+^ T lymphocytes cultured *in vitro* for 20, 44, and 68 hours in the presence of the indicated single drugs or drug combinations. Repeated measures ANOVA with Dunnett’s Multiple Comparison Test was for the statistical analyses (*p <0.05; **p <0.01; ***p <0.001).

Even though ATRA 1 μM and valproic acid 500 and 1000 μM had no or only minor effects on T cell proliferation and viability as well as the CD4:CD8 ratio, both drugs prolonged the expression of CD69 both for CD4^+^ and CD8^+^ T cells, and significantly increased expression was detected after 44 hours (3 healthy individuals tested, Figures 4 and 5). Cytarabine had a similar increasing effect on CD69 expression for both T cell subsets but only when testing the highest concentration (44 μM). The drugs showed additive enhancing effects on CD69 expression both for CD4^+^ and CD8^+^ T cells when testing double or triple combinations, and highly significant differences could then be detected (i) for CD4^+^ T cells when combining ATRA and valproic acid 500 μM; and (ii) especially for CD4^+^ cells but also CD8^+^ T cells when ATRA/valproic acid were combined with low cytarabine concentrations (0.01 and 0.35 μM, p <0.001).

ATRA and valproic acid had opposite effects on CD38 expression by activated T cells; ATRA increased the expression whereas valproic acid decreased the early expression of this marker both for CD4^+^ and CD8^+^ T cells. None of these effects were detected after 68 hours. In contrast, cytarabine did not alter CD38 expression for any concentration tested (0.01, 0.35, 1 and 44 μM). The ATRA-induced enhancement was only maintained in the presence of cytarabine (both for CD4^+^ and CD8^+^ T cells) but not in the presence of ATRA alone. Finally, the valproic acid-induced reduction of CD38 levels was maintained in the presence of cytarabine and the triple combinations did not alter CD38 expression.

Cytarabine 44 μM decreased CD25 expression of CD4^+^ T cells but only after 68 hours of culture (p <0.05). No other single drug or drug combinations altered CD25 expression by activated CD8^+^ and CD4^+^ T cells at any time point tested (data not shown).

### The combination of cytarabine, valproic acid and ATRA reduces the release of FasL and HSP90 but have only weak effects on the release of TRAIL and HSP70 by activated T cells

**Figure 6:**
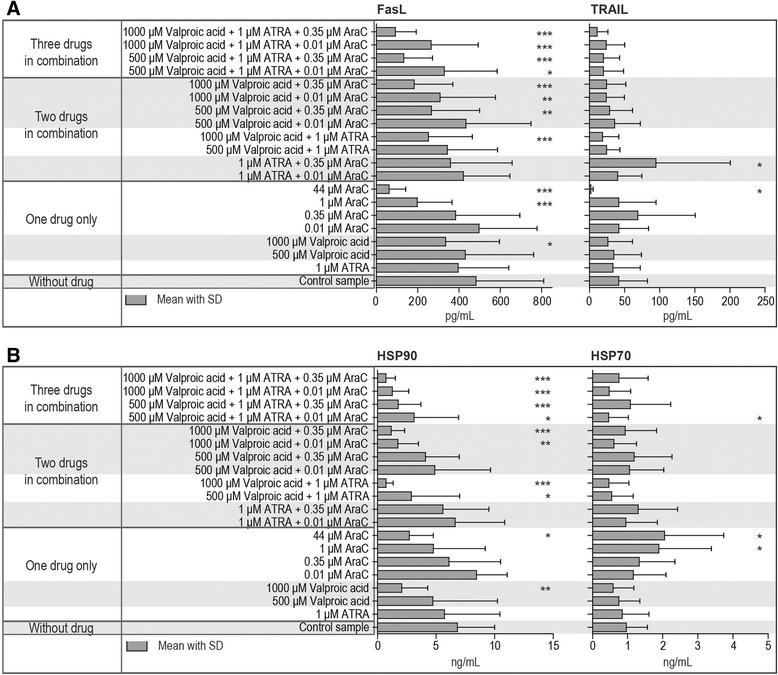
The release of FasL, TRAIL, HSP70 and HSP90 by anti-CD3 plus anti-CD28 activated normal T cells – effects of cytarabine, ATRA and valproic acid tested alone or in combination. The supernatant concentrations of FasL, TRAIL, HSP90 and HSP70 were determined after 4 days of PBMC culture with anti-CD3 plus anti-CD28. Cultures were prepared in medium alone or together with the single drugs or drug combinations indicated in the figures. **(A)** The upper figure presents the FasL (left panel) and TRAIL (right panel) levels, the lower figure **(B)** presents the levels of HSP90 (left panel) and HSP70 (right panel). All results are presented as the mean with SD. Repeated measures ANOVA with Dunnett’s Multiple Comparison Test against control samples were used for statistical analyses (*p <0.05; **p <0.01; ***p <0.001).

Normal PBMC derived from 7 healthy individuals were activated with anti-CD3 and anti-CD28 and cultured for 4 days *in vitro* with or without valproic acid, ATRA or/and cytarabine alone or in combination before supernatant levels of FasL, TRAIL HSP70 and HSP90 were determined (Figure 6). Both valproic acid and cytarabine caused a dose-dependent reduction of HSP90 and FasL. However, the reduction in single-drug cultures was relatively small and the most significant decreases were seen for drug combinations and strong reductions were then seen even when combining drugs at concentrations (cytarabine 0.01 and 0.35 μM, valproic acid 500 μM) that did not cause significant reductions when tested alone. In contrast, the drugs had either no significant or only weak effects on the release of TRAIL and HSP70.

### Only high cytarabine concentrations show a broad inhibitory effect on the cytokine release profile by activated T cell whereas high-level valproic acid as well as combinations of low valproic acid-cytarabine levels inhibits the release of a minor cytokine subset

**Figure 7:**
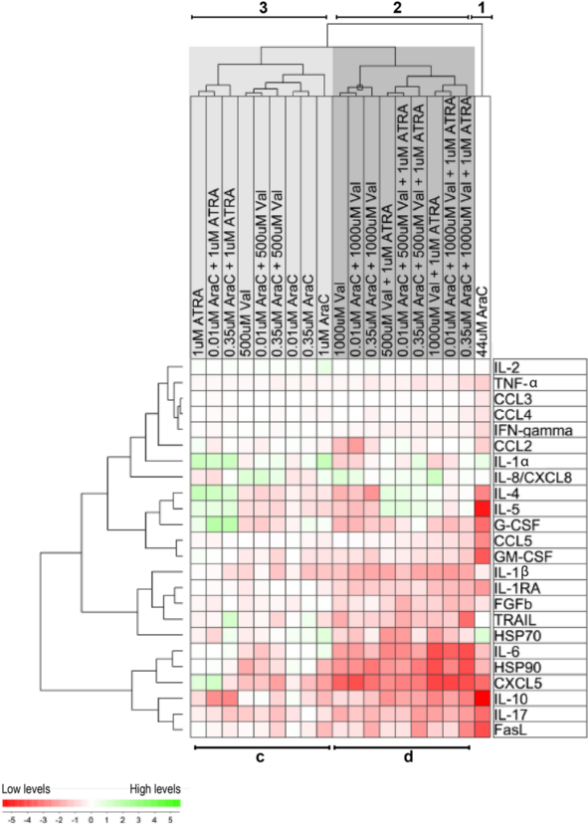
The soluble release profile by activated T cells is altered by valproic acid, ATRA and cytarabine – a two-ways hierarchical cluster analysis. PBMCs derived from 7 healthy individuals were cultured with the T cell activating signal anti-CD3 plus anti-CD28 for 4 days before supernatants were harvested and cytokine levels determined. The cells were then cultured in drugfree control cultures and together with ATRA 1 μM, valproic acid 500 and 100 μM, and cytarabine 0.01, 0.35, 1 and 44 μM, and each drug was also tested in dual or triple combinations as indicated at the top of the figure. The overall results are presented in detail in Additional file 1: Table S1-S4. The results from a two-ways hierarchical cluster analysis including FasL, TRAIL, HSP70 and HSP90 together with 20 cytokines are presented in the figure. We investigated the levels of (i) the immunoregulatory cytokines IFNγ and TNFα; (ii) the growth factors G-CSF, GM,-CSF, VEGF and bFGF; (iii) the chemokines CCL2-5 and CXCL5; and (iv) the interleukins IL1α, IL1β, IL1RA, IL2, IL4-6, IL8, IL10 and IL17. The cytokines formed 4 main clusters as indicated on the left margin of the figure. The analysis included the 19 single drug/drug combinations used throughout the study and the various drugs/drug combinations formed three main clusters.

PBMC derived from 7 healthy individuals were cultured with anti-CD3 plus anti-CD28 for 4 days before supernatant cytokine levels were determined for cultures prepared in medium alone and medium with ATRA 1 μM, valproic acid 500 and 100 μM, and cytarabine 0.01, 0.35, 1 and 44 μM. Each drug was tested alone and in the dual or triple combinations used throughout the study. The overall cytokine results are presented in Additional file 1: Tables S1-S4, and the results are summarized in a two-ways hierarchical cluster analysis including 20 cytokines and in addition FasL, TRAIL, HSP70 and HSP90 (Figure 7). We investigated the levels of (i) the immunoregulatory cytokines IFNγ and TNFα; (ii) the growth factors G-CSF, GM,-CSF, VEGF and bFGF; (iii) the chemokines CCL2-5 and CXCL5; and (iv) the interleukins IL1α, IL1β, IL1RA, IL2, IL4-6, IL8, IL10 and IL17. For this two-way hierarchical cluster analysis the cytokine levels in drug-containing cultures were made relative to the level in the corresponding drugfree control, and the mean relative level of each drug-cytokine combination was used for the analysis that included the 24 soluble mediators and the 19 single drug/drug combinations. The drugs/drug combinations formed three main clusters:

- Only cytarabine 44 μM reduced the levels of a majority of the soluble mediators (Figure 7 right margin, lower and upper middle mediator clusters), including HSP90 as well as several chemokines (CCL5, CXCL5) and immunoregulatory cytokines (IL4-6, IL10, IL17).
- A second cluster was mainly formed by (i) all triple combinations, (ii) the two valproic acid + ATRA combinations and (iii) valproic acid 1000 μM tested alone or in combination with low-level cytarabine. This cluster was characterized by decreased release of the lower mediator cluster similar to cytarabine 44 μM and in addition a relatively weak effect for the lower middle cluster.
- For the other drugs/combinations we only observed weak and divergent effects.

Thus, only high cytarabine levels show a broad and strong inhibition of the T cell cytokine response, whereas high valproic acid levels 1000 μM as well as all triple combinations of low-level cytarabine, low-level valproic acid and ATRA inhibit the release of a defined subset of T cell derived mediators.

### Low cytarabine concentrations show little effect on patient cell viability but significantly reduce their proliferation capability

**Figure 8:**
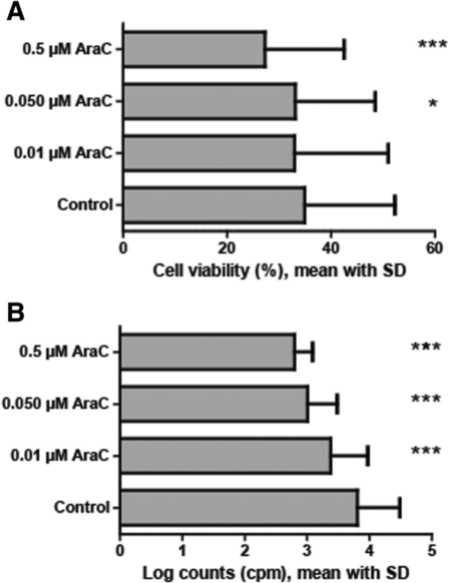
Effects of low-dose cytarabine on the viability and proliferation of primary human AML cells. **(A)** The viability of primary human AML cells after 40 hours of *in vitro* culture in medium alone. Cytarabine seems to have a dose-dependent toxic effect, but the difference reached statistical significance only for the two highest drug concentrations (0.5 and 0.05 μM). **(B)** Low-dose cytarabine showed a dose-dependent antiproliferative effect for the leukemic cells when testing cytokine-dependent AML cell proliferation (medium supplemented with 20 ng/mL of GM-CSF, SCF and Flt3l). The growth inhibition reached statistical significance even when testing cytarabine 0.01 μM. All results are presented as the mean with SD, the pair sample t-test was used for the statistical analyses of the apoptosis data, and the Wilcoxon signed rank test for the proliferation data (*p <0.05; **p <0.01; ***p <0.001).

We investigated the effect of low-dose cytarabine on cell viability and proliferation for AML cells derived from 48 consecutive patients. As described previously the AML cell viability varies widely between patients after *in vitro* incubation due to spontaneous *in vitro* apoptosis [28], and the median viability in our drug-free control cultures was 34% (range 2-64%) in the control samples. We investigated cytarabine 0.5, 0.05 and 0.01 μM (Figure 8A), and the viability was significantly reduced only for the two highest drug concentrations (0.5 μM, median viability 26%, p <0.001; 0.05 μM, median viability 32%, p =0.030). In contrast, all three cytarabine concentrations reduced AML cell proliferation significantly. The median radioactivity corresponded to 5900 counts per minute (cpm) for the drugfree controls (range <1000 – 179200), the activity was only 600 cpm for cytarabine 0.5 μM (p <0.001), 900 cpm for cytarabine 0.05 μM (p <0.001) and 1800 cpm for cytarabine 0.01 μM (p <0.001) (Figure 8B).

## Discussion

Low-toxicity AML-stabilizing treatment is now tried for patients who are unfit for intensive and potentially curative therapy. The HDAC inhibitor valproic acid has been investigated as an antileukemic agent in several clinical studies, usually in combination with ATRA [15] and/or low-dose cytotoxic agents [29]. Three phase II clinical studies have shown that the combination of valproic acid, ATRA and low-dose cytarabine [15,26,27] has low clinical toxicity and can induce complete remission for a minority and disease stabilization for a larger subset of elderly and unfit patients. Despite low clinical toxicity, our *in vitro* results suggest that the treatment may have immunosuppressive effects as we observed decreased T cell viability, reduced T cell proliferation, altered activation-induced expression of membrane molecules and reduced cytokine release (see Table 1 for summary).

**Table 1:** **A summary of pharmacological effects on T cell activation; studies of high cytarabine concentrations alone, low cytarabine levels and low cytarabine levels in combination with ATRA and/or valproic acid**

**Parameter**	**High cytarabine levels (44 and 1.0 μM)**	**Low cytarabine levels (0.35 and 0.01 μM)**	**Low cytarabine levels (0.35 and 0.01 μM) combined with ATRA 1 μM and/or valproic acid (500 and 1000 μM)**
T cell viability	Decreased, apoptosis induction	Decreased only for 0.35 μM	No significant effect for most combinations
T cell proliferation	Decreased	Decreased only for 0.35 μM	A highly significant decrease for combinations with cytarabine 0.35 μM
CD4:CD8 ratio	Decreased	No effect	No effect
CD69 expression	Prolonged expression, especially for CD8^+^ but also CD4^+^ cells	No effect both for CD4^+^ and CD8^+^ cells	CD4^+^ cells: Increased by ATRA and decreased by valproic acid, no effect when combining these two drugs. The ATRA effect maintaining when combined with cytarabine. CD8^+^ cells: Increased by ATRA and valproic acid, the increase was also seen in combinations with cytarabine.
CD38 expression	No effect	No effect both for CD4^+^ and CD8^+^ cells	CD4^+^ cells: Increased by ATRA and decreased by valproic acid, the ATRA effect maintained in the presence of cytarabine. CD8^+^ cells: Increased by ATRA and decreased by valproic acid, the ATRA effect maintained in combinations.
FasL release	Decreased	No effect	Strong decrease in all combinations also with cytarabine 0.01 μM
TRAIL release	Minor decrease only at 44 μM	No effect	Minor effects
HSP90 release	Decreased	No effect	Strong decrease both for cytarabine 0.35 and 0.01 μM
HSP70	Increased	No effect	No effect for all except one combination
Cytokine release; hierarchical cluster analysis including 24 soluble mediators	Decreased levels for two out of 4 main clusters including 9 cytokines, including the cluster showing decreased levels for the drug combinations.	Only minor effects	Decreased levels only for one out of the four main clusters, including 4 cytokines

Cytarabine is commonly used in AML therapy and is then used at single doses ranging from 10 mg/m^2^ up to 3000 mg/m^2^ [30]. The systemic serum levels therefore show a wide variation depending both on the single dose, the administration form and the gender with faster clearance in males [30]. Firstly, the daily doses commonly used in induction treatment of human AML are 100-200 mg/m^2^; the drug can then be given as intermittent injections or as continuous infusions and the clearance is also influenced by the pretreatment leukemia blast count in the blood. The average steady state levels during continuous infusions in one study were 0.4 and 0.8 μM for 100 and 200 mg/m^2^/day, respectively [31]. Levels will be higher when giving these daily doses as injections. Secondly, the drug may also be used at single doses up to 3 g/m^2^ twice daily administered as short-time infusions and resulting in peak concentrations during a 3-hours infusion averaging 50-100 μM [30,31]. Finally, cytarabine is used as low-toxicity subcutaneous injections (10-20 mg/m^2^) once or twice daily usually for a 10 days period with 4-6 weeks intervals [15]; the steady state levels are then below 0.1 μM [30,32-35] and the peak levels after a subcutaneous injection of 10 mg/m^2^/12 hours can be up to 0.2-0.5 μM [18]. In our present study we therefore investigated the concentrations (i) 44 μM that corresponds to peak levels during high-dose treatment; (ii) 1 μM that is reached when using the conventional doses of 100-200 mg/m^2^; and (iii) and 0.35 and 0.01 μM that correspond to levels reached early after and during steady state of low-dose treatment.

Cytarabine effects on T cell activation were concentration-dependent; decreased viability was only seen for the higher concentrations and this is in accordance with previous studies suggesting that cytarabine has cytotoxic effects only at concentrations above 100 nM [30]. However, cytarabine had immunoregulatory effects even at lower level. Several of our present observations suggest that the cytarabine effects on activated T cells at least partly differ between T cell subsets. This is supported both by (i) the altered CD4:CD8 ratio after exposure to high cytarabine levels, (ii) the differences between CD4^+^ and CD8^+^ T cells with regard to cytarabine effects on CD38 and CD69 expression during T cell activation; and (iii) previous studies describing that *in vivo* treatment with our triple combination causes normalization of the increased pre-therapy levels of circulating Treg cells, whereas the levels of circulating Th17 cells are not affected by the treatment [15].

Cytarabine decreased AML cell viability only when tested at 0.5 and 0.05 μM but not at the lowest concentration, whereas the drug inhibited AML cell proliferation even at 0.01 μM. In contrast, T cell proliferation was inhibited only at cytarabine concentrations ≥0.35 μM. Thus, based on the proliferation studies we conclude that primary AML cells are more susceptible to cytarabine than normal T cells; suggesting that there is a therapeutic window for cytarabine treatment that makes it possible to achieve antileukemic effects *in vivo* before severe T cell toxicity occurs.

Our *in vitro* studies showed that valproic acid alone affected T cell activation but only for a minority of our experimental models and usually when testing the highest concentration of 1000 μM that corresponds to a level slightly above the recommended therapeutic serum level [15]. ATRA did not affect T cell activation in most of our studies. However, both drugs contributed to the effects of the triple combination of T cell activation, and in several of our models highly significant immunomodulatory effects were seen only when testing the triple combination.

Early lymphoid reconstitution after intensive and potentially curative antileukemic therapy is associated with decreased risk of later AML relapse, suggesting that post-treatment immunological events are clinically important to maintain disease control and complete hematological remission after end of treatment [36,37]. This association between early lymphoid reconstitution and survival has also been observed after conventional intensive chemotherapy [3], autotransplantation [4] and allotransplantation [5,6]. The mechanisms behind this association are not known [38], but taken together these observations suggest that not only allogeneic but also autologous antileukemic T cell reactivity after intensive treatment or autotransplantation is clinically important.

To the best of our knowledge no previous studies have investigated whether such autologous antileukemic T cell reactivity is important also in patients receiving AML-stabilizing treatment, e.g. the combination of ATRA, valproic acid and cytarabine. There are two major differences between AML patients receiving intensive treatment and patients receiving our low-toxicity triple combination: (i) most patients receiving stabilizing treatment do not achieve complete hematological remission, and (ii) these patients will often receive continued therapy until disease progression. Furthermore, many AML patients have severe leucopenia, including T lymphopenia [16]. During the continued AML-stabilizing treatment most of these patients thus have a combined AML-induced quantitative and treatment-induced qualitative T cell effect. Most of the patients who respond to the treatment do not show increased leukocyte counts [16,39]. If antileukemic immune reactivity is important for the efficiency of AML-stabilizing treatment similar to its effect in patients receiving intensive therapy, the immunosuppressive effects described in our present study may influence the efficiency of this treatment. The question should be addressed in future clinical studies, and one should then especially investigate whether the effect differs between various T cell subsets.

Three clinical studies have previously investigated the antileukemic effect of low-dose cytarabine, ATRA and valproic acid [13,39,40], but the question of immunosuppression was only addressed in one of them [39]. This study showed that even though the drug-induced immunosuppression may be important for the antileukemic efficiency of AML-stabilizing (see above), the triple combination does not seem to have a major impact on the risk of complicating infections in responders to the treatment as these patients usually stayed outside hospital without severe infections as long as the disease was stable without signs of AML progression [39]. However, it should be emphasized that these patients were treated with even a lower cytarabine dose than commonly used for low-dose cytarabine treatment [39], and in contrast to the other two studies ATRA was administered as an intermittent therapy with 2 weeks of treatment at 12 weeks intervals. Our studies suggest that clinically relevant immunosuppression may occur if standard low-dose cytarabine or continuous ATRA therapy is used.

The valproic acid-ATRA-cytarabine combination altered T cell release of several soluble mediators. Firstly, FasL (CD95L) and TNF-related apoptosis-inducing ligand (TRAIL or APO2) may mediate autocrine activation-induced cell death (AICD) to maintain self-tolerance and suppress immune responses [41,42], but soluble CD95L may also induce non-apoptotic signals that promote cell migration [43]. The effects of single drugs and especially our triple combination on FasL release suggest that the treatment may alter cell trafficking. Secondly, the intracellular chaperones HSP90 and HSP70 can be released extracellularly and will then have immunomodulatory effects [44-47]; these effects may be affected by the altered HSP90 levels during T cell activation in the presence of our triple combination. Finally, both cytarabine, valproic acid and ATRA affected the release of several interleukins and chemokines by activated T cells. Taken together these observations suggest that the antileukemic triple combination not only has direct effects on T cells, but probably also indirect effects on other immunocompetent cells mediated by the altered release of soluble mediators during T cell activation.

## Conclusions

Both previous *in vivo* studies and the present *in vitro* studies suggest that the triple combination of low-dose cytarabine, ATRA and valproic acid has immunomodulatory effects, and as discussed above these effects may differ between various T cell subsets. The triple combination has direct effects on the T cells, but it may also affect other immunocompetent cells through the altered release of soluble mediators during T cell activation. This possible risk of immunosuppression should be further investigated in future clinical studies.

## Additional file

Additional file 1: Table S1. Supernatant levels of immunomodulatory cytokines and growth factors. **Table S2.** Supernatant levels of chemokines. **Table S3.** Supernatant levels of Interleukins (IL-1 – IL-5). **Table S4.** Supernatant levels of Interleukins (IL-6 – IL-17).

## Abbreviations

AML: Acute myeloid leukemia
ATRA: All-trans retinoic acid
ELISA: Enzyme-linked immuno-sorbent analyses (ELISA)
HSP: Heat shock protein
PBMC: Peripheral blood mononuclear cells
