# Supplementary material for: Effects of cytarabine on activation of human T cells – cytarabine has concentration-dependent effects that are modulated both by valproic acid and all-trans retinoic acid
Source: BMC Pharmacol Toxicol. 2015 May 2;16:12. doi: 10.1186/s40360-015-0012-2 (PMC4422044; doi:10.1186/s40360-015-0012-2)
Supplement: Additional file 1: Table S1. — Supernatant levels of immunomodulatory cytokines and growth factors. Table S2. Supernatant levels of chemokines. Table S3. Supernatant levels of Interleukins (IL-1 – IL-5). Table S4. Supernatant levels of Interleukins (IL-6 – IL-17). [file 40360_2015_12_MOESM1_ESM.docx]

**Table S1 Supernatant levels of immunomodulatory cytokines and growth factors**

|  | | **IFNγ** | **TNFα** | **G-CSF** | **GM-CSF** | **VEGF** | **FGFbasic** |
| --- | --- | --- | --- | --- | --- | --- | --- |
| **No drugs** | **Control** | **1775 (829-2318)** | **3404 (0-15500)** | **33 (8-72)** | **3648 (2864-9317)** | **0 (0-3)** | **24 (0-46)** |
| **One drug**  **only** | 1 µM ATRA | 1804 (844-2198) | 3450 (1903-15500) | 49 (27-286) | 6261 (2793-10357) | 14 (0-94) | 26 (0-53) |
|  | 500 µM Valproic acid | 1901 (2294-640) | 2508 (2196-15500) | 19 (0-58) | 3013 (2147-7082) | 0 (0-4) | 26 (0-34) |
|  | 1000 µM Valproic acid | 1667 (2494-1022) | 2252 (1243-15500) | 12 (0-71) | 2342 (1835-6816) | 0 (0-12) | 20 (0-23) |
|  | 0.01 µM AraC | 1698 (2494-1022) | 3099 (2764-15500) | 17 (8-63) | 3298 (2937-9871) | 0 (0-7) | 28 (13-31) |
|  | 0.35 µM AraC | 1812 (2064-991) | 2604 (1724-15500) * | 29 (10-111) | 2899 (1962-8666) | 0 (0-0) | 28 (0-44) |
|  | 1 µM AraC | 1810 (798-2313) | 2505 (1495-15500) * | 45 (7-160) | 2268 (1622-8735) | 0 (0-3) | 28 (0-264) |
|  | 44 µM AraC | 606 (44-4591)*** | 916 (260-1777) *** | 5 (0-23) | 362 (52-1556) *** | 9 (0-25) | 10 (0-21) |
| **Two drugs**  **in**  **combination** | 0.01 µM AraC + 1 µM ATRA | 1644 (764-1895) | 2313 (2156-10064) | **47 (31-292)** * | 4429 (2992-10607) | 8 (0-40) | 36 (9-103) |
|  | 0.35 µM AraC + 1 µM ATRA | 1873 (983-2125) | 2629 (1811-1500) | **107 (33-309)** * | 3671 (2585-12144) | 0 (0-7) | 25 (0-43) |
|  | 500 µM Valproic acid + 1 µM ATRA | 1426 (687-1883) | 2103 (1571-15500) | 15 (4-53) | 3046 (1997-10266) | **54 (0-64)** * | 14 (0-30) |
|  | 1000 µM Valproic acid + 1 µM ATRA | 1305 (681-1936) | 1675 (1201-15500) ** | 10 (2-42) | 2294 (1585-7677) | **86 (14-133)** *** | 10 (0-19) |
|  | 500 µM Valproic acid + 0.01 µM AraC | 1886 (1014-2873) | 3074 (0-15500) | 16 (3-99) | 2767 (2581-10214) | 0 (0-0) | 26 (0-39) |
|  | 500 µM Valproic acid + 0.35 µM AraC | 1745 (727-2593) | 2253 (1316-15500) * | 27 (3-103) | 2289 (1481-7132) * | 0 (0-0) | 24 (0-33) |
|  | 1000 µM Valproic acid + 0.01 µM AraC | 1654 (498-2284) | 2189 (1457-15500) | 12 (0-66) | 2442 (1378-7358) | 0 (0-16) | 26 (0-33) |
|  | 1000 µM Valproic acid + 0.35 µM AraC | 1413 (507-2254) | 1675 (1004-15500) ** | 17 (0-86) | 1860 (787-6098) ** | 0 (0-14) | 24 (0-35) |
| **Three drugs**  **in combination** | 500 µM Valproic acid + 1 µM ATRA + 0.01 µM AraC | 1273 (663-2001) | 2048 (1452-15500) | 17 (3-67) | 2780 (2128-10155) | 4 (0-51) | 14 (0-26) |
|  | 500 µM Valproic acid + 1 µM ATRA + 0.35 µM AraC | 1391 (586-2121) | 1512 (0-7924) *** | 31 (4-123) | 2356 (932-9678) | 4 (0-276) | 10 (0-26) |
|  | 1000 µM Valproic acid + 1 µM ATRA + 0.01 µM AraC | 1249 (718-2084) | 1610 (1381-15500) * | 13 (2-59) | 2340 (1621-8273) | **45 (0-98)** ** | 0 (0-24) |
|  | 1000 µM Valproic acid + 1 µM ATRA + 0.35 µM AraC | 1228 (522-1847) * | 1486 (819-4007) *** | 12 (2-64) | 1678 (749-6715) * | **33 (7-339)** * | 9 (0-16) |

The table shows the supernatant levels of cytokines (median and range) for activated T cells cultured for 4 days with or without the indicated drugs (n=7).All cytokine concentrations are presented as pg/mL. Repeated measures ANOVA with Dunnett's Multiple Comparison Test was used to determine statistically significant differences. Red denotes statistically significant decreased levels compared to controls. Green denotes statistically significant increased levels compared to control. * denotes p <0.05. ** denotes p <0.01. *** denotes p <0.001.

**Table S2 Supernatant levels of chemokines**

|  | | **CCL2** | **CCL3** | **CCL4** | **CCL5** | **CXCL5** |
| --- | --- | --- | --- | --- | --- | --- |
| **No drugs** | **Control** | **2160 (1054-3903)** | **8964 (8230-9075)** | **5390 (4842-6321)** | **10009 (3407-10587)** | **359 (116-1244)** |
| **One drug**  **only** | 1 µM ATRA | 2487 (1912-3469) | 8889 (7943-9061) | 5337 (4056-6596) | 8774 (2843-11277) | **782 (166-2101)** ** |
|  | 500 µM Valproic acid | 2481 (631-3562) | 8942 (8182-9069) | 5346 (4671-6358) | 9571 (4836-10620) | 118 (49-393) |
|  | 1000 µM Valproic acid | 1015 (397-2326) | 9008 (8250-9098) | 5412 (4472-6428) | 9828 (2600-11015) | 52 (9-153) * |
|  | 0.01 µM AraC | 1614 (873-3107) | 8368 (7942-8986) | 5443 (4428-6600) | 8015 (7095-11202) | 230 (108-1168) |
|  | 0.35 µM AraC | 1776 (1112-2676) | 8795 (7301-8966) | 5002 (4395-6535) | 8133 (1676-10674) | 138 (15-741) |
|  | 1 µM AraC | 1920 (643-4085) | 8455 (8173-9098) | 4786 (3762-6535) | 8606 (1557-10563) | 205 (18-359) ** |
|  | 44 µM AraC | 892 (98-3505) * | 6480 (467-8874) *** | 3798 (669-4304) *** | 1689 (182-3227) *** | 42 (10-124) ** |
| **Two drugs**  **in**  **combination** | 0.01 µM AraC + 1 µM ATRA | 1674 (1560-2851) | 8014 (7380-8983) | 4588 (4254-5853) | 8049 (6365-8767) | **836 (344-1876)** * |
|  | 0.35 µM AraC + 1 µM ATRA | 2546 (1966-3288) | 8907 (8092-9088) | 5154 (4257-6418) | 9312 (2445-10513) | 244 (54-947) |
|  | 500 µM Valproic acid + 1 µM ATRA | 2678 (1379-3080) | 8859 (7315-9017) | 5293 (3399-6341) | 8967 (4919-10255) | 85 (43-184) * |
|  | 1000 µM Valproic acid + 1 µM ATRA | 2228 (956-4026) | 8816 (6450-9008) | 5081 (2950-6041) * | 6860 (1694-10598) | 21 (10-42) ** |
|  | 500 µM Valproic acid + 0.01 µM AraC | 2278 (860-4391) | 8950 (8252-9053) | 5371 (4785-6532) | 9443 (3886-11623) | 127 (48-490) |
|  | 500 µM Valproic acid + 0.35 µM AraC | 2146 (1025-3676) | 8921 (7895-9068) | 4999 (3994-6286) | 9743 (1908-10793) | 85 (23-288) * |
|  | 1000 µM Valproic acid + 0.01 µM AraC | 743 (198-2028) * | 8956 (8248-9085) | 5399 (3875-6456) | 10119 (2617-10904) | 31 (8-138) ** |
|  | 1000 µM Valproic acid + 0.35 µM AraC | 1295 (440-2593) | 8886 (6441-9142) | 5295 (3427-5386) * | 8968 (1158-10804) | 46 (10-120) ** |
| **Three drugs**  **in combination** | 500 µM Valproic acid + 1 µM ATRA + 0.01 µM AraC | 2785 (1533-3390) | 8884 (7584-8991) | 5312 (3746-5912) | 8152 (2584-10453) | 47 (20-152) * |
|  | 500 µM Valproic acid + 1 µM ATRA + 0.35 µM AraC | 2250 (1259-3702) | 8811 (6066-8975) | 4558 (3271-5957) * | 7486 (745-10141) * | 51 (16-151) * |
|  | 1000 µM Valproic acid + 1 µM ATRA + 0.01 µM AraC | 1652 (898-2860) | 8829 (7934-8994) | 5071 (3707-5989) | 6407 (2176-10121) | 20 (10-62) ** |
|  | 1000 µM Valproic acid + 1 µM ATRA + 0.35 µM AraC | 2405 (1293-3096) | 8666 (5229-9053) | 4177 (2993-5753) ** | 5518 (744-9008) * | 30 (5-66) ** |

The table shows the supernatant levels of cytokines (median and range) for activated T cells cultured for 4 days with or without the indicated drugs (n=7).All cytokine concentrations are presented as pg/mL. Repeated measures ANOVA with Dunnett's Multiple Comparison Test was used to determine statistically significant differences. Red denotes statistically significant decreased levels compared to controls. Green denotes statistically significant increased levels compared to control. * denotes p <0.05. ** denotes p <0.01. *** denotes p <0.001.

**Table S3 Supernatant levels of Interleukins (IL-1 – IL-5)**

|  | | **IL-1α** | **IL-1β** | **IL-1RA** | **IL-2** | **IL-4** | **IL-5** |
| --- | --- | --- | --- | --- | --- | --- | --- |
| **No drugs** | Control | 43 (8-77) | 111 (17-332) | 3123 (379-8500) | 1974 (7-2281) | 9 (0-65) | 1114 (362-1248) |
| **One drug**  **only** | 1 µM ATRA | 79 (15-235) | 170 (17-302) | 3591 (664-8500) | 1752 (12-2344) | **39 (8-415)** ** | **1428 (1026-1912)** * |
|  | 500 µM Valproic acid | 51 (10-122) | 106 (26-111) | 2216 (382-8500) | 1689 (1053-2269) | 10 (6-28) | 674 (581-746) |
|  | 1000 µM Valproic acid | 22 (11-126) | 32 (20-88) ** | 1778 (299-3137) * | 1493 (957-2159) | 8 (0-21) | 457 (203-608) *** |
|  | 0.01 µM AraC | 33 (10-82) | 127 (20-196) | 2817 (796-8500) | 1366 (863-2127) | 12 (8-49) | 981 (563-1102) |
|  | 0.35 µM AraC | 43 (14-178) | 79 (11-126) | 3427 (961-8500) | 1750 (601-2269) | 9 (0-35) | 744 (187-1173) |
|  | 1 µM AraC | **167 (16-489)** * | 75 (10-173) * | 3061 (499-8500) | **1852 (1305-5152)** * | 17 (0-60) | 545 (74-1125) * |
|  | 44 µM AraC | 84 (12-423) | 83 (11-376) | 651 (138-4098) *** | 1437 (830-1939) | 9 (0-29) | 29 (3-164) *** |
| **Two drugs**  **in**  **combination** | 0.01 µM AraC + 1 µM ATRA | **82 (28-264)** * | 199 (44-277) | 2912 (761-4650) | 1795 (362-2326) | 51 (18-317) | **1458 (1250-1907)** * |
|  | 0.35 µM AraC + 1 µM ATRA | 87 (33-285) | 122 (19-190) | 2829 (784-8500) | 1689 (32-2360) | 26 (5-100) | **1785 (1258-1905)** ** |
|  | 500 µM Valproic acid + 1 µM ATRA | 43 (13-101) | 44 (13-56) ** | 1568 (336-4362) | 2017 (1744-2334) | 28 (7-94) | **1858 (1288-1892)** ** |
|  | 1000 µM Valproic acid + 1 µM ATRA | 13 (9-50) | 14 (10-52) *** | 1209 (219-2067) * | 1973 (1724-2153) | 16 (0-51) | 1434 (454-1768) |
|  | 500 µM Valproic acid + 0.01 µM AraC | 35 (15-222) | 66 (39-190) | 2965 (474-8500) | 2146 (1177-2262) | 6 (0-34) | 609 (438-754) * |
|  | 500 µM Valproic acid + 0.35 µM AraC | 81 (12-310) | 68 (7-119) * | 2730 (416-8500) | 1806 (1181-2234) | 6 (0-20) | 597 (125-774) ** |
|  | 1000 µM Valproic acid + 0.01 µM AraC | 22 (7-148) | 29 (10-86) ** | 1921 (218-3590) | 1363 (1243-2160) | 8 (0-17) | 443 (272-603) ** |
|  | 1000 µM Valproic acid + 0.35 µM AraC | 46 (9-293) | 35 (5-74) ** | 2227 (325-8500) | 1634 (714-2175) | 6 (0-13) | 423 (40-705) *** |
| **Three drugs**  **in combination** | 500 µM Valproic acid + 1 µM ATRA + 0.01 µM AraC | 47 (10-105) | 42 (11-88) ** | 1800 (286-4516) | 1983 (1946-2290) | 31 (12-109) | **1873 (1031-1905)** * |
|  | 500 µM Valproic acid + 1 µM ATRA + 0.35 µM AraC | 68 (7-232) | 46 (5-71) ** | 2259 (243-8500) | 1783 (308-2318) | 30 (0-60) | 1678 (69-1875) |
|  | 1000 µM Valproic acid + 1 µM ATRA + 0.01 µM AraC | 26 (8-51) | 25 (8-81) ** | 1162 (238-3339) * | 1900 (1641-2185) | 13 (6-602) | 859 (540-1831) |
|  | 1000 µM Valproic acid + 1 µM ATRA + 0.35 µM AraC | 30 (8-136) | 19 (6-58) *** | 1438 (236-8500) * | 1654 (914-2192) | 20 (0-51) | 1223 (100-1726) |

The table shows the supernatant levels of cytokines (median and range) for activated T cells cultured for 4 days with or without the indicated drugs (n=7).All cytokine concentrations are presented as pg/mL. Repeated measures ANOVA with Dunnett's Multiple Comparison Test was used to determine statistically significant differences. Red denotes statistically significant decreased levels compared to controls. Green denotes statistically significant increased levels compared to control. * denotes p <0.05. ** denotes p <0.01. *** denotes p <0.001.

**Table S4 Supernatant levels of Interleukins (IL-6 – IL-17)**

|  | | **IL6** | **IL8** | **IL10** | **IL17** |
| --- | --- | --- | --- | --- | --- |
| **No drugs** | Control | 877 (271-1904) | 4201 (0-20824) | 719 (135-1425) | 380 (54-1401) |
| **One drug**  **only** | 1 µM ATRA | 820 (331-1613) | 4632 (2434-47000) | 441 (59-833) * | 176 (115-2818) |
|  | 500 µM Valproic acid | 552 (59-1688) | 4520 (1139-46372) | 551 (126-817) | 126 (34-472) * |
|  | 1000 µM Valproic acid | 196 (16-1485) | 6242 (3309-15273) | 555 (80-644) | 114 (38-465) * |
|  | 0.01 µM AraC | 411 (231-1971) | 4906 (1956-14668) | 390 (351-814) | 256 (125-1216) |
|  | 0.35 µM AraC | 688 (303-2693) | 2586 (2199-5312) | 284 (182-605) * | 340 (137-1540) |
|  | 1 µM AraC | 1173 (226-2691) | 5663 (2614-15114) | 182 (92-315) ** | 168 (50-937) |
|  | 44 µM AraC | 526 (63-1660) | 2985 (1044-9530) | 5 (1-42) *** | 24 (14-233) *** |
| **Two**  **drugs**  **in**  **combination** | 0.01 µM AraC + 1 µM ATRA | 669 (270-1368) | 2002 (1120-5240) | 123 (54-463) ** | 143 (69-650) |
|  | 0.35 µM AraC + 1 µM ATRA | 1122 (317-1859) | 3818 (2654-13753) | 101 (45-353) ** | 118 (63-585) |
|  | 500 µM Valproic acid + 1 µM ATRA | 135 (35-336) | 4097 (1808-47000) | 129 (27-324) ** | 60 (25-579) * |
|  | 1000 µM Valproic acid + 1 µM ATRA | 35 (10-167) * | 7112 (1383-28445) | 116 (4-193) *** | 52 (23-391) * |
|  | 500 µM Valproic acid + 0.01 µM AraC | 335 (74-3076) | 5138 (0-12812) | 577 (203-890) | 180 (45-619) |
|  | 500 µM Valproic acid + 0.35 µM AraC | 588 (89-2419) | 5052 (953-8734) | 378 (74-579) * | 147 (53-678) |
|  | 1000 µM Valproic acid + 0.01 µM AraC | 193 (15-1710) | 7900 (1026-13551) | 257 (74-709) | 94 (26-539) * |
|  | 1000 µM Valproic acid + 0.35 µM AraC | 332 (25-1898) | 5214 (976-11209) | 168 (19-677) * | 73 (36-456) |
| **Three**  **drugs**  **in**  **combination** | 500 µM Valproic acid + 1 µM ATRA + 0.01 µM AraC | 143 (26-461) | 3715 (2289-6642) | 123 (60-242) ** | 83 (45-453) * |
|  | 500 µM Valproic acid + 1 µM ATRA + 0.35 µM AraC | 262 (36-880) | 5049 (0-11784) | 100 (36-406) ** | 72 (36-440) * |
|  | 1000 µM Valproic acid + 1 µM ATRA + 0.01 µM AraC | 163 (16-233) | 4396 (3384-15025) | 107 (35-749) * | 44 (18-533) * |
|  | 1000 µM Valproic acid + 1 µM ATRA + 0.35 µM AraC | 55 (17-415) * | 5183 (4022-7037) | 77 (20-390) *** | 51 (20-320) *** |

The table shows the supernatant levels of cytokines (median and range) for activated T cells cultured for 4 days with or without the indicated drugs (n=7).All cytokine concentrations are presented as pg/mL. Repeated measures ANOVA with Dunnett's Multiple Comparison Test was used to determine statistically significant differences. Red denotes statistically significant decreased levels compared to controls. Green denotes statistically significant increased levels compared to control. * denotes p <0.05. ** denotes p <0.01. *** denotes p <0.001.
